# Plasma neurofilament light chain: A biomarker predicting severity in patients with acute ischemic stroke

**DOI:** 10.1097/MD.0000000000029692

**Published:** 2022-06-30

**Authors:** Jixia Wu, Daqing Wu, Youbao Liang, Zhen Zhang, Lei Zhuang, Zhaoping Wang

**Affiliations:** a Department of Neurology, Bengbu First People’s Hospital, Bengbu, Anhui Province, China; b Department of Finance, Bengbu Energy Group Co., Ltd., Bengbu, Anhui Province, China; c Department of Laboratory, Bengbu First People’s Hospital, Bengbu, Anhui Province, China.

**Keywords:** biomarker, ischemic stroke, neurofilament light chain, plasma, predicting, prognosis

## Abstract

Neurofilament light chain (NfL) levels have proved to be a good biomarker in cerebrospinal fluid (CSF) correlating with the degree of neuronal injury and neurodegeneration. However, little is known about the value of plasma neurofilament light chain (pNfL) levels in predicting the clinical prognosis of patients with acute cerebral infarction. This study aimed to explore whether pNfL could be used as a biomarker to predict the severity of the outcomes of acute ischemic stroke (AIS).

Patients with AIS were included from the Department of Neurology of the First People’s Hospital of Bengbu City from January 2018 to May 2019, as well as health control (HC). The plasma levels of NfL in patients with AIS (n = 60) at 2 days, 7 days, and 6 months after stroke, as well as in HCs (n = 60) were measured by electrochemiluminescence immunoassay(ECL) on the Meso Scale Discovery platform. Stroke severity was analyzed at admission using the National Institutes of Health Stroke Scale score. Functional outcomes were assessed at different times using the modified Rankin Scale (mRS) and Barthel Index.

The mean level of pNfL in patients with ischemic stroke (IS) at 2 days (225.86 pg/L) after stroke was significantly higher than that in HC (107.02 pg/L) and gradually increased 7 days after stroke (316.23 pg/L) (*P* < .0001). The mean level of pNfL in patients with IS at 6 months after stroke was 173.38 pg/L, which was still significantly higher than that of HC. The levels of pNfL at 7 days after stroke independently predicted modified Rankin Scale scores (mRS) (*R* = 0.621, *P* < .001), Barthel Index (*R* = –0.716, *P* < .001), and National Institutes of Health Stroke Scale (*R* = –0.736, *P* < .001). The diagnostic severity and prognosis were evaluated by ROC curve, an area under the receiver operator curve of 0.812 (*P* = .001, 95% CI: 0.69–0.93) at 7 days.

Plasma NfL levels reflect neuronal injury after AIS. It changes with time and has a certain relationship with prognosis and may be a promising biomarker for predicting the severity of neuroaxonal injury in patients with acute IS.

## 1. Introduction

Stroke is becoming a leading cause of morbidity worldwide. Because of its effect on physical function, it is association with an increased risk of cognitive impairment and dementia,^[1–4]^ stroke can lead to physical disability, lasting brain damage, or death. Stroke will be one of the most significant global public health problems.

A stroke usually occurs when a blood vessel that carries oxygen and nutrients to the brain is either blocked by a clot or bursts (or ruptures) resulting in insufficient blood and oxygen supply to part of brain, which leads to its damage. Although clinical scales provide insight into stroke severity, they are often crude noncontinuous measures of the extent of tissue injury. There is thus a clear need for biomarkers that provide a continuous measure of the degree of neuroaxonal injury regardless of whether the stroke is ischemic or hemorrhagic, and such biomarkers have the potential to demonstrate improved stroke trial efficiency and the ability to predict clinically meaningful outcomes like poststroke cognitive impairment and response to rehabilitation.^[5]^ Monitoring pNfL may provide an indication for the extent of brain damage during the stroke process.^[6]^

pNfL is the main structural component of the intermediate filaments in neurons, which is composed of 4 different protein subunits, including central nervous system-specific α-internexin, pNfL chain, medium chain, and heavy chain.^[7,8]^ These proteins are synthesized in the body of the neuron and assembled into intermediate filaments in the axon, where they provide stability.^[9]^

Many studies indicate that high levels of pNfL in CSF have been found in different neuroinflammatory and neurodegenerative diseases, such as dementia,^[10]^ Alzheimer’s disease (AD),^[11]^ Parkinsonian disorders,^[12]^ and amyotrophic lateral sclerosis.^[13]^ The level of pNfL is higher in AD patients than healthy populations and is also associated with disease progression.^[14]^ Recent studies show that high plasma levels of NfL are found after acute damage to the brain and its levels correlate with the severity of damage^[6,15,16]^ and higher blood NfL levels in the acute phase after stroke predict unfavorable outcomes. Blood NfL is also used for the evaluation for the functional improvement in the late phase after stroke.^[17–20]^ Although pNfL has been widely used for above conditions, reports on high levels of pNfL as a biomarker of acute ischemic stroke (AIS) are few and inconsistent.

This study aimed to explore the evolution of temporal patterns in pNfL concentrations after AIS. Furthermore, we explored the association of pNfL levels with severity and BI of AIS patients and its clinic application as a promising biomarker for the long-term prognosis of stroke in patients with AIS.

## 2. Material and Methods

### 2.1. Subjects

#### 2.1.1 Patients

Sixty admitted patients with AIS were included between January 2018 and May 2019 from the First People’s Hospital of Bengbu City. All the patients were diagnosed and routinely evaluated by computed tomography (CT) and magnetic resonance imaging (MRI). Sixty health control (HC) subjects were included by physical examination. Ischemic strokes were classified according to the original TOAST and MRI results at admission. They were divided into lacunar infarction and large atherosclerotic cerebral infarction groups.

#### 2.1.2 Exclusion criteria

The following exclusion criteria included other causes of dementia, psychiatric diseases, coma, aphasia, and severe neurological impairment.

#### 2.1.3 Ethical

Ethical approval for the study was obtained from the Bengbu First People’s Hospital Ethics Committee. Before inclusion, informed consent was obtained from each subject.

### 2.2. Blood measurements

#### 2.2.1 The pNfL measure

Blood samples were taken from patients with AIS at 2 days, 7 days, and 6 months after admission, and at the same times for the HC group. Venous blood was collected in 4 mL vacutainers and EDTA tubes. The collected blood was centrifuged at 2000 g for 10 minutes, plasma samples aliquoted and stored at −80°C freezer until analysis. There were no freeze-thaw cycles and the temperature of the freezers was monitored, verifying an unbroken freezing time.

The levels of pNfL in controls and ischemic stroke patients were measured using an electrochemiluminescence (ECL) immunoassay in the Meso Scale Discovery (MSD) platform from MSD. ECL usually took only 4–5 hours, after which the sample was briefly incubated for 2 hours. Antibody was incubated for 2 hours before detection. The sensitivity of ECL was as high as 0.05 pg/mL, and the effective linear range was as high as 6 log, which could simultaneously take into account the detection of high and low abundance proteins. This technology improved the analytical sensitivity to an extent that reliable quantification of pNfL levels in plasma became possible across the range of concentrations in disease and physiological conditions.

#### 2.2.2 Homocysteine and cystatin measure

The levels of homocysteine (Hcy) and cystatin (Cys) were also measured in the sample of patients with AIS as well as HC. The fasting venous blood were collected in the morning after admission and sent to the laboratory department of our hospital to detect the serum homocysteine and cystatin with homocysteine and cystatin C Kit and Olympus 5800 automatic biochemical instrument.

### 2.3. Neuropsychological assessment

All the patients were evaluated by National Institutes of Health Stroke Scale (NIHSS) at 2 days, 7 days, and 6 months after admission. Performance in activities of daily living of all the patients was scored with the Barthel Index (BI). The Modified Rankin Scale (mRS) was used to measure the outcomes of functional recovery after stroke.

The BI has scores ranging from 0 to 100, where scores of 100 indicate self-care; scores above 60 indicate basic selfcare; scores from 60 to 40 indicate that some assistance is needed; scores from 40 to 20 indicate that great assistance is needed; scores below 20 indicate complete dependency. Functional recovery was assessed using the mRS ranging from 0 (asymptomatic) to 5 (severe disability). Unfavorable functional outcome was defined as a mRS score of 3 to 5.

### 2.4. Data analysis

All data were analyzed using SPSS 23.0. Descriptive statistics were used to summarize clinical and demographic data. The means of variables between patients with AIS and HC were compared by independent sample t-test. We tested correlations between pNfL and neurological function assessment using Pearson correlations. Analysis of paired t-test was used to observe the variance of pNfL at day 2, day 7, and month 6. We quantified the discriminative ability of pNfL for distinguishing between IS cases and controls using area under the receiver operator curve (AUC). All tests were 2-sided. Statistical significance was set at *P* < .05.

## 3. Results

### 3.1. Demographics and clinical characteristics

Significant differences of hypertension, hyperlipidemia, and diabetes mellitus, but not age and gender were identified between patients with AIS and HC (*P* < .001, Table 1). Significant differences were also found for plasma Hcy and pNfL, NIHSS, mRS, and BI (Table 1).

**Table 1:** Demographics and clinical characteristics.

Variable	Control (n = 60)	IS (n = 60)	*P*
Age, mean (SD), y	67.26 (11.2)	68.17 (9.12)	.246
Female (%)	24 (40)	19 (31.6)	.468
Hyperlipidemia (%)	3 (5)	18 (30)	.0001
Diabetes (%)	3 (5)	22(36.7)	.011
Hypertension history (%)	6 (10)	40 (66.7)	.0001
Hcy, mean (SD)	10.25 (7.79)	18.32 (5.68)	.001
Cys, mean (SD)	3.87 (15.64)	4.88 (6.65)	.872
pNfL, mean (SD)	105.80 (45.68)	225.86 (194.71)	.001
NIHSS, mean (SD)	1 (2.67)	4.21 (3.47)	.001
mRS, mean (SD)	0	2.38 (1.35)	.0001
BI, mean (SD)	100	70.51(27.89)	.0001

pNfL for baseline at day 2, *P* ≤ .05; *P* ≤ .0001.

BI = Barthel Index; Cys = cystatin; Hcy = homocysteine; mRS = Modified Rankin scale; NIHSS = National Institutes of Health Stroke Scale; pNfL = plasma neurofilament light chain at day 2.

### 3.2. Correlation between Cys and ischemic stroke patients

Cys levels in ischemic stroke patients were comparable to HCs, Cys levels did not correlate to AIS.

### 3.3. Correlation between Hcy and ischemic stroke patients

Hcy is the basis of infarction stroke and atherosclerosis.^[21]^ In our analysis, Hcy levels in ischemic stroke patients were comparable to HCs and Hcy levels did correlate to AIS.

### 3.4. Plasma NfL

**Figure 1.**
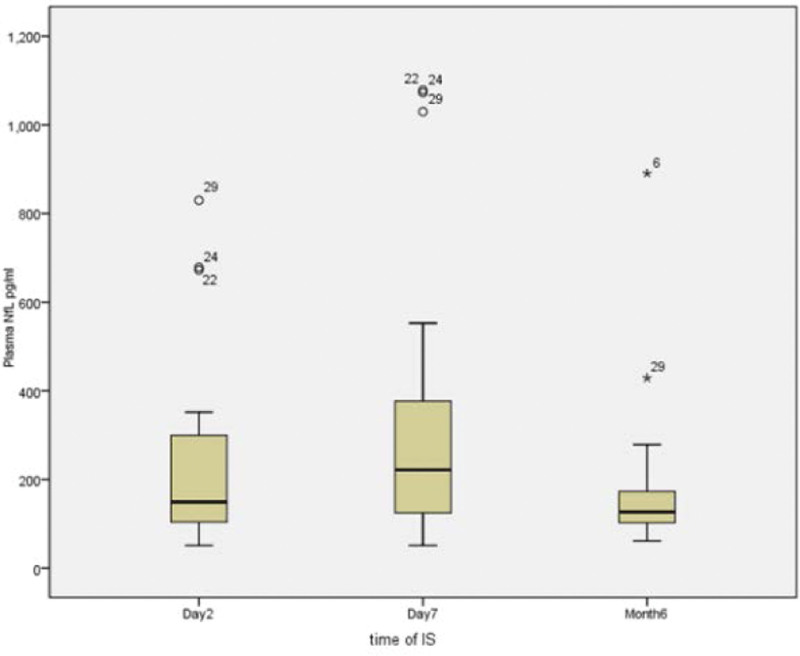
Content of plasma NfL at different time after acute ischemic stroke.

The mean level of pNfL in patients with AIS at day 2 after stroke were significantly higher than HC (225.86 vs 105.8 pg/L, *P* < .001), which reached the peak at day 7 after stroke (316.23 pg/L), significantly higher than that of both day 2 and month 6 (173.38 pg/L, *P* < .001). Additionally, the mean levels of pNfL in patients with AIS both at day 7 and month 6 after stroke were significantly higher than HC. Figure 1 clearly shows the difference of NFL at 3 different times after stroke (Table 2).

**Table 2:** The pNfL mean levels at different time after stroke.

Times	pNfL, mean (pg/L)	*P*
2 D	225.86	.001
7 D	316.23	.001
6 M	173.38	.001

Significant differences were pNfL mean levels comparable to healthy controls at different time after stroke.

D = day; M = month.

### 3.5. Correlation between plasma pNfL and neurological assessment

**Figure 2.**
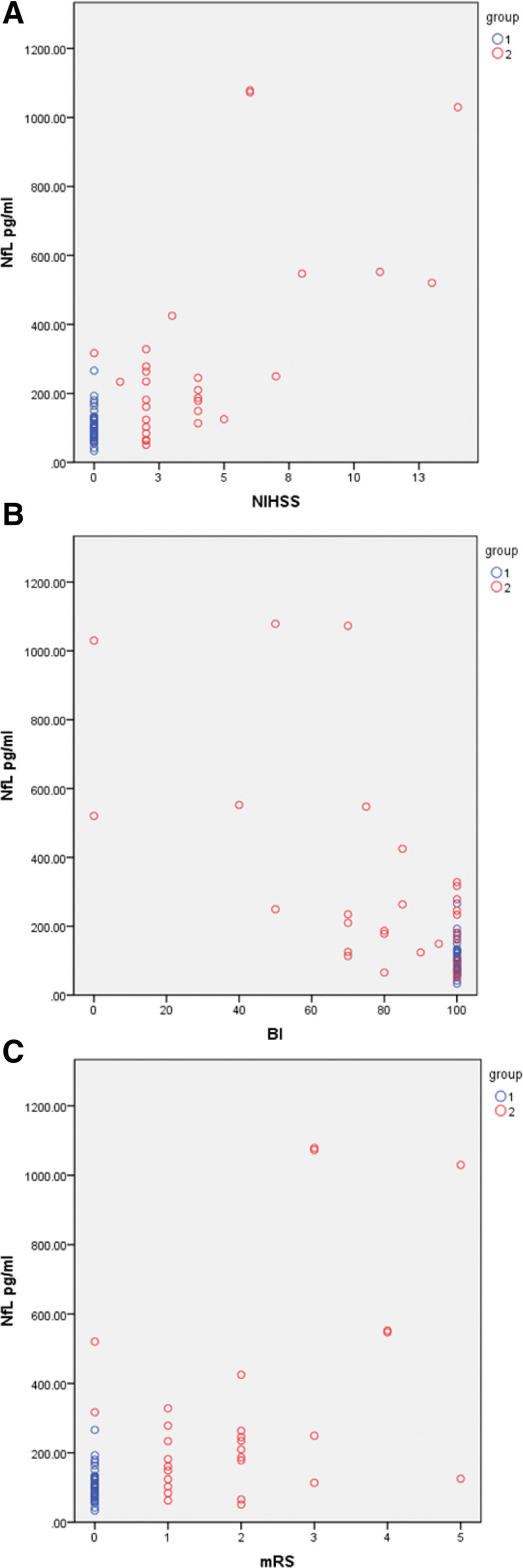
Plasma pNfL at day 7 correlated to BI2, NIHSS, mRs. (A) Association of plasma pNfL and NIHSS at day 7; (B) Association of plasma pNfL and BI at day 7; (C) Association of plasma NfL and mRs at day 7. blue is controls; red is IS. BI = Barthel Index; mRS = Modified Rankin scale; NIHSS = National Institutes of Health Stroke Scale; pNfL = plasma neurofilament light chain.

The pNfL of patients with IS were significantly negatively associated with BI scores at day 2 (*R* = –0.647, *P* ≤ .001), day 7 (*R* = –0.716, *P* ≤ .01, Fig. 2B), and month 6 (*R* = –0.685, *P* ≤ .001) after stroke. These results indicate that IS patients with higher levels of pNfL have lower BI scores and poorer ability of daily living.

The pNfL of patients with IS were significantly positively associated with NIHSS in patients IS at day 2 (*R* = 0.715, *P* < .05), day 7 (*R* = 0.736, *P* ≤ .05) (Fig. 2A), and month 6 (*R* = 0.716, *P* ≤ .05) after stroke. These results indicate that IS patients with higher levels of pNfL have sustained more serious nerve injury.

The pNfL of patients with IS were significantly negatively associated with modified Rankin’s scores at day 2 (*R* = –0.605, *P* ≤ .001), day 7 (*R* = –0.621, *P* ≤ .01, Fig. 2C), and month 6 (*R* = –0.557, *P* ≤ .001) after stroke. These results indicate that IS patients with higher levels of pNfL have worse recovery of neurological function and prognosis. The content of pNfL in large atherosclerotic cerebral infarction was significantly higher than that in lacunar infarction, and it was still significantly higher than that in lacunar infarction after 6 months. BI score was significantly lower than lacunar infarction, modified Rankin’s score was significantly lower, and NIHSS was also higher. These results indicate that pNfL levels can be used to judge the severity and prognosis of stroke patients.

### 3.6. Diagnostic effect of pNfL

**Figure 3.**
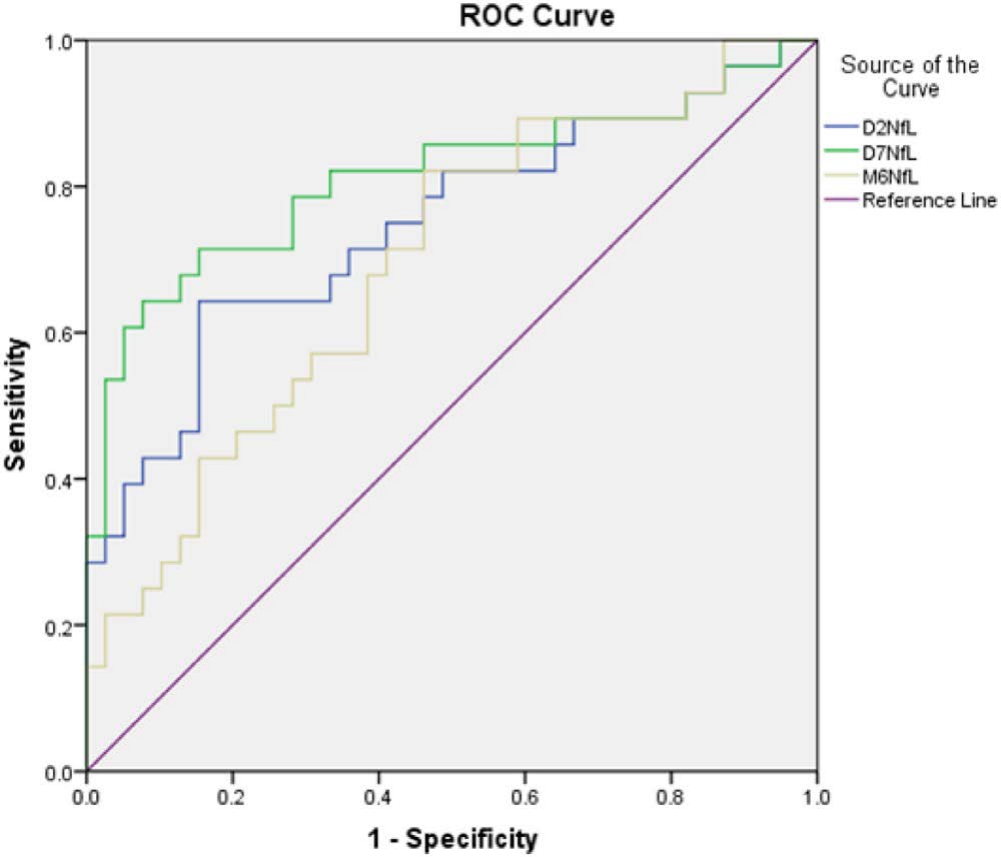
Area under the receiver operator curve at 3 occasions. Sensitivity and specificity analysis of pNfL. *P* < .05 for day 2 and day 7, *P* < .0001 for M6. AUC1 = 0.746; AUC2 = 0.812; AUC3 = 0.694; M6 = plasma PNFL at month 6. AUC = area under the receiver operator curve.

The optimal cutoff value of pNfL concentration as an indicator for auxiliary diagnosis of moderate-to-high stroke (moderate-to-high stroke was defined as a NIHSS ≥5) was assessed by ROC curve. The optimal

threshold was 133.37 pg/mL, which yielded a sensitivity of 64.3% and a specificity of 84.6%, AUC of 0.746 (*P* = .002, 95% CI: 0.60–0.87) at day 2; the optimal threshold was 173.38 pg/mL, which yielded a sensitivity of 64.3% and a specificity of 93%, AUC of 0.812 (*P* = .001, 95% CI: 0.69–0.93) at day 7, and the optimal threshold was 100.33 pg/mL, which yielded a sensitivity of 82.1% and a specificity of 54%, AUC of 0.694 (*P* = .023, 95% CI: 0.53–0.81) at month 6. These results indicate that pNfL has the best diagnostic effect at 3 time after stroke. The diagnostic and predictive value was better on day 7 (Fig. 3).

## 4. Discussion

NfL is a promising diagnostic and prognostic fluid biomarker with high-clinical value in many neurological disorders.^[22–24]^ Owing to its convenience and lesser invasiveness in clinical practice, blood NfL measurement has been an exciting and active field of research.^[25]^ In this study, we confirmed that pNfL may be used as a biomarker to predict the severity of neuroaxonal injury in patients with AIS. We found that pNfL levels are significantly higher in AIS patients than in HCs; the pNfL levels in AIS patients correlated with stroke severity (NIHSS score) at admission and with functional outcome (BI and mRS score) 6 months poststroke. This is line with previous studies that the peak level of pNfL was found at 3 weeks after stroke, with its level recovering to normal after 3–5 months.^[7]^ Wang P et.al. showed that high pNfL concentration was associated with clinical severity at admission, and a poor early functional prognosis in patients with AIS.^[25]^ Nielsen et al^[26]^ also found that pNfL levels was well related with severity on admission and functional outcome at 3 months. Moreover, we found that the levels of Hcy levels increased acutely in the blood of ischemic stroke patients compared with HCs. However, we did not observe any change in Cys mechanistically, the role of pNfL in pathophysiology of stroke might be through the following pathways. At the early stage immediately after ischemia, neurons are unable to sustain their normal transmembrane ionic gradient and homoeostasis. This elicits several processes that lead to cell death: excitotoxicity, oxidative and nitrative stress, inflammation, and apoptosis. These pathophysiological processes are seriously injurious to neurons, glia, and endothelial cells.^[27,28]^ Part of the brain tissue (core) undergoes irreversible neuronal damage due to necrotic cell death, whereas the surrounding tissues contain salvageable and metabolically active cells (penumbra), in which cell death occurs less rapidly.^[29]^ During the early stage, the level of pNfL slightly increases. However, with the prolongation of ischemia time, the injury of nerve cells increased in severity and the number of dead cells increased, the necrosis and liquefaction of the lesion brain tissue is released into the peripheral blood. Then the level of pNfL gradually increases to its peak at 7 days to 3 weeks after stroke.^[30,31]^ Functional recovery is most pronounced in the first 6 months after the stroke event.^[32]^ The liquefied necrotic brain tissue is cleared by lattice cells, whereas brain tissue atrophy, glial scar formation in small lesions, and apoplexy sac formation in large lesions are identified, so the level of pNfL is not normalized after 6 months.

Comparable findings of neurofilament dynamics have been reported in other studies.^[33,34]^ Prolonged release of pNfL into the blood after acute neuronal injury might be caused by persistent blood-brain barrier breakdown, but ongoing postischemic immunological or inflammatory processes could also explain these findings.^[15]^

In patients with acute IS, the level of pNfL is strongly associated with NIHSS, mRs, and BI at 2 days, 7 days, and 6 months after stroke. Patients with higher level of pNfL had lower BI score and poorer ability of daily living, which is consistent with NIHSS score. Furthermore, we found that patients with poor functional outcomes within 6 months after stroke showed have higher pNfL concentration at admission than those with good outcomes. This indicates that pNfL level is strongly associated with stroke severity at admission.

We demonstrated that the level of pNfL chain was significantly elevated in an acute IS cohort (*P* < .0001) with an ROC of 0.809 at day 7. Thus pNfL is a sensitive and specific circulating measure, and it is a promising diagnostic biomarker for acute cerebral ischemia.^[6,33]^ Additionally, we also observed that homocysteine in peripheral blood was closely related to acute IS, which was consistent with previous studies.^[21,22]^ The combination of Hcy and pNfL could increase the reliability of diagnoses.

Under normal conditions, neurofilaments are highly stable within axons, and their turnover is low. Many, if not all, pathological processes that cause axonal damage release neurofilament proteins into the extracellular fluid, CSF, and peripheral blood, depending on the extent of the damage. High levels of neurofilaments, therefore, are general indicators of axonal damage irrespective of its cause and any clinical diagnosis, where blood levels of neurofilaments are useful for not only monitoring and predicting progression in various acute and chronic neurological diseases but also assessing the efficacy and toxicity of treatment.^[16]^

However, this study has several limitations. First, the sample size of the study was small. Second, the relationship between pNfL and ischemic volume was not examined. Moreover, We could not determine whether the changes in plasma levels reflect in CSF.

## 5. Conclusion

In summary, our study showed that pNfL may be a promising biomarker for the neuroaxonal injury and severity of patients with acute IS. Further large scale studies are warranted to validate the value of pNfL as a biomarker of IS in clinical diagnostics.

## Acknowledgments

Thank you to the staff of department of Neurology and the Laboratory of the First People Hospital of Bengbu City for their assistance in collecting and storing specimens.

## Author contributions

Conceptualization: Jixia Wu, Daqing Wu, Youbao Liang, Lei Zhuang. Data curation:Jixia Wu, Zhen Zhang, Zhaoping Wang. Supervision: Jixia Wu. Writing-original: Jixia Wu. Writing – review and editing: Jixia Wu, Daqing Wu.
